# Feasibility of AI and Human Standardized Patients to Enhance Customer Discovery Communication Skills in Medical Students: Preliminary Evaluation of an Observational Cohort Study

**DOI:** 10.2196/95225

**Published:** 2026-09-10

**Authors:** Nathaniel Hafer, Monika Chitre, Melissa Fischer, Julie LeMoine

**Affiliations:** 1 University of Massachusetts Chan Medical School Worcester, MA United States; 2 My Diji Helpers, Inc Watertown, MA United States

**Keywords:** undergraduate medical education, generative artificial intelligence, GenAI, artificial intelligence, AI, simulation training, academic success, entrepreneurship, interview, I-corps

## Abstract

**Background:**

Medical students must build skills beyond traditional clinical domains to best shape the evolving health care system and fill a variety of professional roles after graduation. AI offers new methods for medical education.

**Objective:**

This study aimed to develop and evaluate the feasibility and preliminary effectiveness of traditional (human) standardized patient (SP) engagement and generative AI (GenAI)–generated SP engagement as a low-stakes way for students to practice customer discovery interviews.

**Methods:**

An interactive classroom experience was created in the UMass Chan Medical School interprofessional Center for Experiential Learning and Simulation (iCELS) using an observational cohort design to simulate 2 different interview scenarios with human SPs and AI-generated SPs. The simulation with human SPs was conducted with 74 first-year medical students in the Entrepreneurship, Biodesign, and Innovation Pathway starting in 2023. In 2026, 2 GenAI chatbot interview agents, using OpenAI GPT-5.2, were developed and added as an additional part of the simulation experience. The 2 scenario-specific GenAI agents (patient and clinician/physician) used structured instructions defining their roles, interview contexts, knowledge boundaries, response styles, time limits, feedback, and scoring rubric. The simulations took place over a single 2-hour session in the spring semester. Interview scenarios, sample questions and answers, and an evaluation rubric were developed for both SPs to promote consistency. Primary outcomes were student attitudes regarding the acceptability of the simulation and self-efficacy, collected via a validated online survey immediately after class. Students were asked to rate statements on a 3-point (1=“not at all relevant,” 2=“somewhat relevant,” and 3=“very relevant”), 4-point (1=“strongly disagree” to 4=“strongly agree”), or 5-point (1=“strongly disagree,” 3=“neutral,” and 5=“strongly agree”) Likert scale and were also allowed to provide open-ended comments.

**Results:**

The students gave the simulation with human SPs high scores, with 85% (23/27) agreeing or strongly agreeing that the exercise met learning objectives, with a median score of 4 (IQR 1) on a 4-point Likert scale. Responses to the AI chatbot session had a bimodal distribution; for example, 65% (13/20) of students agreed that “This experience with an AI chatbot SP helped me practice my customer discovery interview skills” while 35% (7/20) disagreed with this statement. A Mann-Whitney *U* test compared responses between the human SP and AI chatbot groups and revealed no significant difference in responses.

**Conclusions:**

This preliminary study with an AI chatbot was able to replicate realistic customer discovery interviews in 2 different scenarios. Larger studies are needed to determine the feasibility and acceptability of these chatbots for practicing customer discovery skills. Future work will create transcripts of both the AI chatbot and human SP interviews and use independent raters to score interview quality based on our scoring rubric. Finally, we plan to enhance the chatbots to provide a more immersive and realistic experience for students.

## Introduction

With the emergence of AI, particularly generative AI (GenAI), new learning methods are available for the delivery of medical education [1-4]. One popular approach uses AI as a personalized, virtual teaching assistant for students. A recent study from the University of Toronto showed a correlation between AI teaching assistant use and improved examination scores and student engagement [5]. AI has also been shown to help medical students with their literature searches and scientific writing skills [6]. Another approach is to use AI as virtual standardized patients (SPs) to teach clinical communication skills in a repeatable, low-pressure format. For instance, investigators have reported using GenAI chatbots to assist students in communication skill development and objective structured clinical examination (OSCE) preparation [7-10]. A study of Caribbean medical students used AI SPs to practice clinical diagnostic skills verbally. Faculty observers noted that students used more structured reasoning and asked better questions after practicing with the virtual SP. Students also expressed feeling more confident and organized in their clinical reasoning [11]. Recently, the use of AI has been extended to dental trainees. An AI SP simulation with dental residents increased their confidence in managing difficult discussions and received high user experience ratings [12].

In addition to objectively improving learning outcomes, students also report familiarity with and use of AI in their medical education. Semistructured interviews with medical students reported frequent use of ChatGPT to summarize topics, clarify complex concepts, develop sample examination questions, and summarize research. Students described large language models (LLMs) as complementary to traditional materials such as course notes and textbooks [13]. A recent study of English-speaking medical students in Canada reported that 96.5% of respondents had used at least 1 LLM [14]. Approximately 63.6% of students rated LLMs as somewhat or much better than traditional learning methods. Interestingly, only about 25% of respondents reported monthly or more frequent use of LLMs to assist with OSCE preparation.

There is an ongoing need for medical students to build skills beyond traditional clinical areas to shape the evolving health care system and fill a variety of professional roles after graduation. Examples include training in engineering, leadership, and the humanities. Medical schools are adding new topics to their curricula, such as product development and entrepreneurship. These programs often incorporate elements of Biodesign or I-Corps, which heavily rely on customer discovery interviews to discover end-user needs [15]. Customer discovery is the process of talking to people to determine the commercial viability of a new product or technology. Customer discovery interviews, especially with more senior clinicians and executives, can be particularly intimidating for students since they usually have little experience with this skill. Our aim was to develop and evaluate the feasibility and preliminary effectiveness of traditional (human) SP engagement and GenAI-generated SP engagement as a low-stakes way for students to practice customer discovery interviews.

This study is grounded in the Kolb experiential learning theory, which uses experience, reflection, conceptualization, and experimentation to drive effective learning. This model has been successfully used in medical education to provide a safe, low-risk space for students to practice communication skills, clinical decision-making, and innovation and entrepreneurship [16-18]. Best practices for how to use AI for customer discovery have been suggested [19]; however, there is a lack of experiential opportunities to improve medical student learning and self-efficacy in innovation and entrepreneurship programs. This is especially true regarding the development of skills for conducting successful customer discovery interviews. Our hypothesis was that a learning simulation with an AI chatbot would be equally acceptable to students as an experience with human SPs. We also hypothesized that the AI chatbot experience and human SP experience would perform equally well with respect to students’ self-reported perceptions of learning and self-efficacy. The ultimate goal of this work is to build a scalable product that will enhance medical student confidence and increase the quality of information collected via interviews.

## Methods

### Overview

We used an observational cohort study design with 74 first-year medical students at UMass Chan Medical School. Students enrolled in the Entrepreneurship, Biomedical Innovation, and Design Pathway participated in the study as part of their first-year curriculum. Completion of the evaluative surveys was optional. The simulation exercise was conducted over a single 2-hour session in the UMass Chan interprofessional Center for Experiential Learning and Simulation (iCELS). After a brief introduction, students were split into 4 groups that rotated through each of the customer discovery scenarios (Table 1). Students were instructed to spend about 15 minutes per interview, with 5 to 10 minutes for feedback. At the conclusion of all 4 sessions, student attitudes and self-efficacy were surveyed using electronic data capture tools (Multimedia Appendix 1). Survey results were analyzed using Microsoft Excel 365 to determine the median and IQR. A Mann-Whitney *U* test was conducted using Statistics Kingdom software [20].

**Table 1:** Simulation day class schedule.

Time	Activity	Human patient: cardiovascular disease	Human physician: diabetes care	AI patient: cardiovascular disease	AI physician: diabetes care
1:00-1:05 PM	Introduction	—^a^	—	—	—
1:05-1:30 PM	Simulation 1	Cohort A	Cohort B	Cohort C	Cohort D
1:30-1:55 PM	Simulation 2	Cohort D	Cohort A	Cohort B	Cohort C
1:55-2:20 PM	Simulation 3	Cohort C	Cohort D	Cohort A	Cohort B
2:20-2:45 PM	Simulation 4	Cohort B	Cohort C	Cohort D	Cohort A
2:45-3:00 PM	Debrief and class survey	—	—	—	—

^a^Not applicable (all student cohorts were together in 1 group for the intro and debrief and class survey).

### Simulation Experience

An interactive classroom experience using an observational cohort study design was developed in the UMass Chan Medical School iCELS, consisting of 2 customer discovery interview simulations: 1 involving an SP portraying a patient and a second involving an SP portraying a health care provider. Each simulation was conducted using 2 delivery modalities: traditional (human) SP engagement and GenAI-generated SP engagement. Our goal was to familiarize students with the process of conducting interviews. Strong oral communication skills are an essential part of conducting successful interviews. Conducting unbiased interviews is a critical part of customer discovery and allows individuals to test different hypotheses about a business model.

The customer discovery interview simulations with traditional (human) SPs were conducted with first-year medical students in the Entrepreneurship, Biodesign, and Innovation Pathway starting in 2023. The simulation was repeated with first-year students in 2024 and 2025. In 2026, speech-enabled AI chatbots were added as an additional part of the simulation experience. A total of 74 students participated: 10 in 2023, 20 in 2024, 20 in 2025, and 24 in 2026.

Two customer discovery scenarios (Textbox 1), sample questions and answers (Multimedia Appendix 2), and an evaluation rubric (Multimedia Appendix 3) were developed to simulate a common experience. To prepare for the simulation, human SPs reviewed the materials and practiced the scenarios with the course instructor (NH). SPs and students were also provided with a series of customer discovery videos that described best practices and common pitfalls for interviews [21].

**Clinical scenarios used to help students practice clinical and customer discovery interview skills.**

**Scenario A**

You are a student working with a faculty mentor who is developing a software app to help patients monitor their cardiovascular disease symptoms at home between visits to the physician. You are interviewing *patients with cardiovascular disease* who may benefit from using this app. You want to understand how they currently manage their disease, what types of technology they use at home, how they communicate with their provider, and if they would be comfortable using technology to monitor, report, and act on their health condition.

**Scenario B**

You are a student working with a faculty mentor who is developing a patient-provider messaging system for patients with diabetes. You are interviewing *physicians who may use this system*. You want to understand how they currently manage patients, what types of technology they use in their clinic, how they currently communicate with their patients, and if they would be comfortable using technology to remotely monitor, report, and act on their patients’ health conditions.

### Speech-Enabled AI Simulation

The GenAI interview agents were developed and tested between November 2025 and February 2026. GPT-5.2 (OpenAI) was the LLM used during these interventions in February 2026. OpenAI’s GPT chatbot was selected because it was the most commonly reported GenAI tool used by medical students in a separate institutional AI ethics study. GPT-5.2 was the current OpenAI model version available to the study team at the time of the intervention, making it a familiar and feasible choice for this low-stakes GenAI interview practice.

The speech-to-text and text-to-speech service provider was OpenAI. Text and speech interactions were implemented through the GPT Builder (OpenAI) voice-enabled interface rather than a custom API. Specific speech service, temperature, sampling, and generation parameter version numbers were not exposed in the no-code GPT Builder interface and were therefore not independently configured or recorded by the study team. The GPT configuration enabled web search and canvas image generation, whereas code interpreter or data analysis and custom actions were not enabled or used.

Learners first engaged with either the patient or the physician or clinician. Next, during the feedback phase, the agents exited these assigned patient or physician roles and used the same rules, rubrics, and scoring structures used by the human SPs to assess learner performance and offer feedback. Learners engaged with the agents on Windows 11 (Microsoft Corp)–based desktop or laptop computers equipped with headsets and microphones to support voice interaction. Each workstation was configured with direct URL access to the appropriate GPT agent corresponding to the assigned simulation scenario.

Consistency safeguards included the same case-specific instructions across learners, direct adaptation from the human SP guidance materials and evaluation rubric, defined roles and knowledge boundaries, standardized timing rules, and the same rubric-based feedback logic used by the human SPs. The agents were iteratively reviewed during the months prior to the intervention by the project team, course instructors, and subject matter experts, and by the human SP training and coordination team. This review process focused on agent persona, behavioral expectations, case constraints, rubric translation, and whether the agents engaged with learners in a manner closely aligned with the same activity delivered by the human SP. No formal testing protocol or findings were documented. Full agent instructions, including the rubric logic, are provided in the appendix documents to support transparency and replication of this intervention (Multimedia Appendices 3 and 4).

Following completion of the interview, the agent used the canvas capability to display a written transcript of the interaction along with a written version of the structured feedback based on the rubric that was provided via speech. All learner interactions with the GenAI SP agents occurred within the secure iCELS simulation environment, with iCELS staff present to provide technical oversight and support during the activity. The agents were configured using prompt-based and rule-based logic; no additional training was performed. Configuration materials, including prompts and rubric logic, were documented to support reproducibility of the intervention (Multimedia Appendix 4).

### Methodological Safeguards

Safeguards were incorporated to maintain a focused and pedagogically appropriate learning experience. Constraint-based agentic logic was embedded within the agent instructions for each GPT to limit the scope of the GenAI SP responses and prevent the agent from drifting into topics outside the intended customer discovery interview scenario. These constraints ensured that learner interactions remained aligned with the instructional objectives and the defined interview framework. This design approach preserved pedagogical oversight by ensuring that faculty-defined learning goals and rubric expectations governed the interaction rather than allowing unrestricted conversational behavior.

Additionally, all learner interactions with the GenAI SP agents occurred within the secure iCELS simulation environment. iCELS staff were present during the classroom sessions to provide technical oversight and support, ensuring that the activity was conducted within a supervised and controlled educational setting.

### Data Collection

Student attitudes and self-efficacy were surveyed at the conclusion of all 4 sessions using Qualtrics (Qualtrics Inc) or REDCap (Veritas Health Innovation) electronic data capture tools (Multimedia Appendix 1) [15,22-24]. Students were asked to rate statements on a 3-point (1=“not at all relevant,” 2=“somewhat relevant,” and 3=“very relevant”), 4-point (1=“strongly disagree”-4=“strongly agree”), or 5-point (1=“strongly disagree,” 3=“neutral,” and 5=“strongly agree”) Likert scale and were also allowed to provide open-ended comments.

### Data Analysis

Survey results were analyzed using Microsoft Excel to determine the median and IQR. A Mann-Whitney *U* test was conducted using Statistics Kingdom software [20].

### Ethical Considerations

The UMass Chan Institutional Review Board (IRB) determined that this work was not human subjects research (IRB STUDY00002795). Participation in the simulation and surveys was voluntary. Individuals were not compensated for participating.

## Results

The results of the 2023 and 2024 surveys are shown in Table 2. Across these 2 years, a total of 30 students participated in the simulation, of whom 27 (90%) completed the voluntary survey. Most students (23/27, 85%) said that the effectiveness of the human SP session in meeting the stated learning objectives was good or excellent, with a median score of 4 (IQR 1) on a 4-point Likert scale. The other questions were also rated highly: 100% (27/27; median 3, IQR 1 on a 3-point Likert scale) said that the exercise was somewhat or very relevant to the Pathway’s goals, 96% (26/27; median 3, IQR 1 on a 3-point Likert scale) agreed that the simulation was somewhat or very effective in enhancing knowledge, and 89% (24/27; median 3, IQR 0.5 on a 3-point Likert scale) said that the simulation was somewhat or very relevant to their longitudinal project.

**Table 2:** Evaluation of pooled survey responses from 2023 and 2024 simulations with human standardized patients.

Responses	How effective was this session in meeting the stated learning objectives? (n=27), n (%)	How relevant was this information to your Pathway goals? (n=27), n (%)	How effective was this activity in enhancing your knowledge? (n=27), n (%)	Was this information relevant to your Pathway’s Longitudinal Project? (n=27), n (%)
1. Poor or not relevant at all	1 (4)	0 (0)	1 (4)	3 (11)
2. Fair, somewhat relevant, or somewhat effective	3 (11)	9 (33)	9 (33)	4 (15)
3. Good, very relevant, or very effective	8 (30)	18 (67)	17 (63)	20 (74)
4. Excellent	15 (55)	N/A^a^	N/A	N/A
Median (IQR)	4 (1)	3 (1)	3 (1)	3 (0.5)

^a^N/A: not applicable.

**Figure 1:**
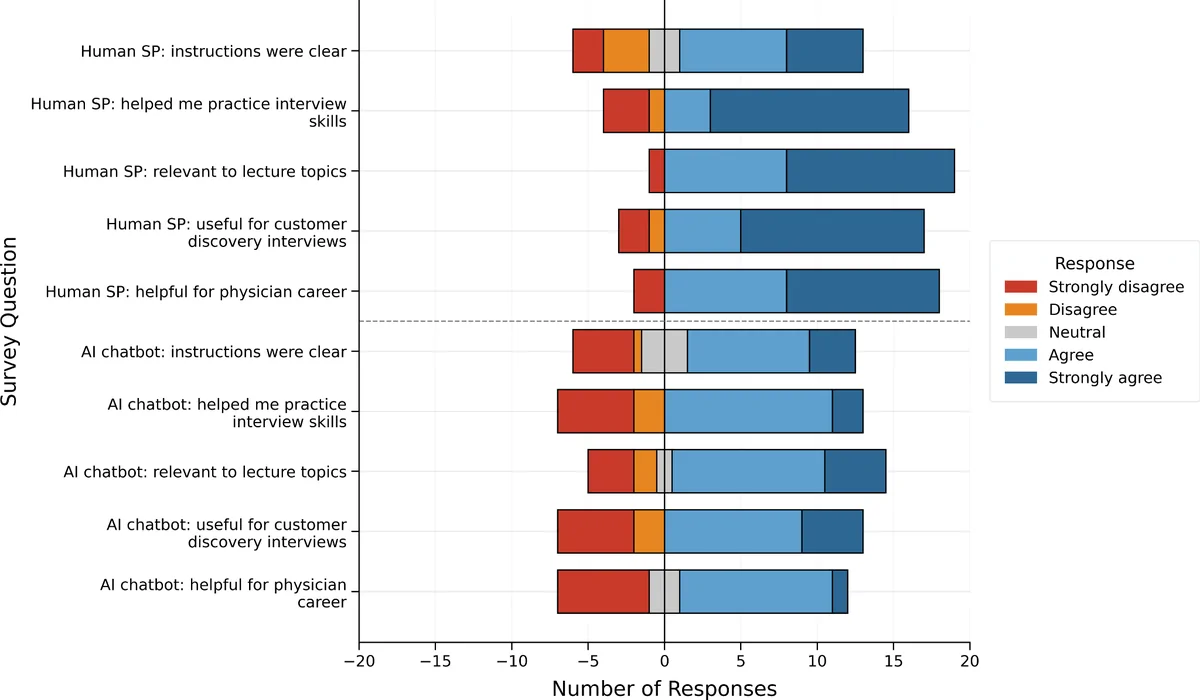
Diverging stacked bar chart showing responses to the survey questions presented in Table 4. Response categories are shown as strongly disagree, disagree, neutral, agree, and strongly agree. SP: standardized patient.

In 2026, the student survey was expanded to include questions about the human SP experience and the AI chatbot experience. Of the 24 students who participated in the experience, 20 completed the voluntary survey. Even though the questions were worded differently, student responses to the human SP experience were generally positive (Table 3). Of the 20 respondents, 12 (60%) agreed or strongly agreed with the statement, “the interview instructions were clear and easy to follow,” and 16 (80%) to 19 (95%) of responses agreed or strongly agreed with the other 4 questions. Interestingly, the student responses to the AI chatbot experience showed a bimodal distribution, with 55% (11/20) to 70% (14/20) of respondents agreeing or strongly agreeing with the question statements and 25% (5/20) to 35% (7/20) of respondents disagreeing or strongly disagreeing with the statements. Figure 1 represents the data as a diverging stacked bar chart. A Mann-Whitney *U* test was conducted to compare results between the human SP and AI chatbot groups. Results revealed no significant difference between the groups for each question, as reported at the bottom of Table 3. When prompted to give open-ended feedback about the experience, respondents commented on the organization of the session and technical details related to account setup, which unfortunately posed challenges for some learners. Notably, 1 student said the following:

I was not the biggest fan of chatting with the AI bot. I found it repeated the same thing with different words a lot. Overall I think chatting with an AI sim is somewhat overwhelming and dystopian.

**Table 3:** Evaluation of 2026 simulations with human standardized patients (SPs) and AI chatbots.

Response options				“The interview instructions were clear and easy to follow.”		“This experience helped me practice my customer discovery interview skills.”		“This experience was relevant to topics discussed in lecture.”		“I can think of a way to use my experience in my own customer discovery interviews.”		“The interview experience was helpful and can be applied to my career as a physician.”
**Human SP experience**												
	**Participants (n=20), n (%)**											
		1. Strongly disagree	2 (10)		3 (15)		1 (5)		2 (10)		2 (10)	
		2. Disagree	4 (20)		1 (5)		0 (0)		1 (5)		0 (0)	
		3. Neutral	2 10		0 (0)		0 (0)		0 (0)		0 (0)	
		4. Agree	7 (35)		3 (15)		8 (40)		5 (25)		8 (40)	
		5. Strongly agree	5 (25)		13 (65)		11 (55)		12 (60)		10 (50)	
	Likert scale score, median (IQR)			4 (2.3)		5 (1)		5 (1)		5 (1)		4.5 (1)
**AI chatbot experience**												
	**Participants (n=20), n (%)**											
		1. Strongly disagree	4 (20)		5 (25)		3 (15)		5 (25)		6 (30)	
		2. Disagree	2 (10)		2 (10)		2 (10)		2 (10)		1 (5)	
		3. Neutral	3 (15)		0 (0)		1 (5)		0 (0)		2 (10)	
		4. Agree	8 (40)		11 (55)		10 (50)		9 (45)		10 (50)	
		5. Strongly agree	3 (15)		2 (10)		4 (20)		4 (20)		1 (5)	
	Likert scale score, median (IQR)			4 (2)		4 (2.3)		4 (1.3)		4 (2.3)		4 (3)
Mann-Whitney *U* test				11		12.5		9.5		11.5		11
*P* value				.83		.92		.60		.92		.83
*r* value				0.067		0.033		0.17		0.033		0.067

We analyzed written feedback for a more nuanced understanding of these critiques. Among the 9 students who rated ≥2 AI chatbot questions with a score of 3 (neutral response) or lower (disagree or strongly disagree), 6 provided written feedback (Table 4). We classified 4 critiques as technical in nature and 3 as pedagogical, suggesting that these problems contributed roughly equally to user dissatisfaction.

**Table 4:** Written feedback from students who responded to ≥2 AI chatbot questions with a score of ≤3.

ID	Comment summary	Category
9	N/A^a^	—^b^
10	“It was a bit difficult to get the AI to give feedback.”	Technical
12	N/A	—
18	N/A	—
19	“Ran out of voice time; not sure what we were to discuss.”	Technical; pedagogical
21	“Not realistic and not helpful with human communication”	Pedagogical
22	“Chatbot repeated comments, overwhelming and dystopian.”	Pedagogical
23	“AI was unresponsive, did not respond properly.”	Technical
26	“User did not log into account which limited chatbot capabilities.”	Technical

^a^N/A: not applicable.

^b^Not available.

## Discussion

### Principal Findings

GenAI offers the opportunity for educators to approach medical education in new ways. In this report, we describe how GenAI can be used to train students in customer discovery interviews in a low-stakes simulated setting. Students practiced their interview technique with human SPs and GenAI SPs. In this preliminary study, survey responses suggested that both groups were equally acceptable to students. We also observed that the GenAI experience and human SP experience performed equally well in terms of self-reported perceptions of learning and self-efficacy. Written feedback provided a mix of technical and pedagogical concerns with the GenAI simulation, similar to those reported in other published reports [1-4,11]. In the future, we envision that this work can extend existing work to collect medical histories [6-8] and facilitate patient recruitment into clinical trials. We envision using a framework, such as that described by Nguyen et al [25], to ground our future work on how to use AI as a learning tool.

### Challenges

As this is a preliminary study, it is important to note the limitations of this work. First, the simulation with the GenAI SP has only been tested on a small sample of first-year medical students at a single medical school. The simulation was designed for students in the Entrepreneurship, Biomedical Innovation, and Design Pathway, so it is unknown how this simulation would perform or be perceived in the general student population. As this is the first study of our GenAI tool to help students practice customer discovery interviews, we do not yet have information about the reproducibility and generalizability of our work. Future studies with additional, larger medical student cohorts both at UMass and at other institutions could help to address the limitations of our current work.

Several operational challenges, technical challenges, and agentic logic adjustment requirements emerged during implementation. Use of GPT-5–based agents with advanced voice capabilities introduced service limitations related to use minutes. During the live instructional event, the voice service exhausted available minutes, interrupting interactions midsession and reverting from the high-quality voice interface to a more robotic fallback voice. Access to the project’s custom GPTs also required participants to use OpenAI accounts, which created additional access challenges during setup. Due to these technical challenges, some learners completed the experience on personal devices. It is unclear how directly these technical challenges may have impacted learner responses to the postexperience surveys; however, we hypothesize that overcoming these challenges will improve acceptance and satisfaction. A challenge in persona design arose because the voice used in the advanced voice interface is determined by the desktop or device rather than by the GPT configuration itself. This prevented consistent assignment of persona-specific voices across participants.

Finally, coordinating learner engagement between human SP encounters and GenAI-based SP encounters simultaneously introduced event complexity. Although both modalities used equivalent time limits and the same assessment rubrics, differences in conversational dynamics required active facilitation to keep the learning experiences aligned.

Changes to the agentic logic for each GPT agent were required to reduce the overly cheerful nature of responses and the repeated use of words such as “absolutely” when starting responses. Several iterations of the rubric used were required to adjust the feedback so that it was pedagogically consistent with the instructors’ goals. This, in turn, required updates to the human SP rubric information. Some of these challenges can be partially mitigated with changed licensing and aggressive setup testing. However, the former can greatly affect costs. These items together point to the need for more extensive testing and validation of the LLM. It is important to factor the planned duration for exchanges in this type of use case prior to establishing the event plan, as engagement time can be reset daily based on the type of license.

### Plans for the Future

In the future, we plan to incorporate demographic information into our learner surveys to see if the negative opinions about the GenAI experience are associated with particular groups. We will also include questions about user technology acceptance to see how this is associated with opinions about the GenAI chatbot simulation.

To date, we have only used these speech-enabled GenAI chatbots with medical students. In the future, we will test these simulations with additional learners, including participants in our I-Corps programs, who include company founders, students, and senior faculty. We plan to collect transcripts of both the speech-enabled GenAI chatbot and human SP interviews. We will score interview quality based on our rubric using independent raters. Other investigators have shown that the performance of AI chatbots is comparable to that of human interviewers, so we hypothesize that we will observe similar results [6-8].

Finally, we plan to enhance the speech-enabled GenAI chatbots to provide a more immersive and realistic experience for students. For instance, we will add avatars to the interviewees so that learners have a visual component to interact with, provide avatars with different personality types to mimic difficult conversations, and enable students to practice with more than 2 scenarios. iCELS has established a GenAI-based complex conversation platform, GenAI Conversational Avatar Practice System, that enables learner engagement with GenAI avatars. Translating and expanding this speech-enabled use case to fully embodied avatars is the next planned step (under review).

### Conclusions

A speech-enabled GenAI chatbot was created to help students practice customer discovery interviews in 2 different scenarios. Our preliminary work suggests that the human SPs and the speech-enabled GenAI SP simulation are acceptable to students and may improve self-efficacy. Future work will further demonstrate the feasibility and validity of using chatbots as a method for medical students to practice their customer discovery communication skills.

## Appendix

Multimedia Appendix 1 Student survey.

Multimedia Appendix 2 Standardized patient sample interview questions and answers.

Multimedia Appendix 3 Customer discovery interview practice rating rubric.

Multimedia Appendix 4 Agentic definition document for each of the 2 GPTs.

## Abbreviations

GenAI: generative AI
iCELS: interprofessional Center for Experiential Learning and Simulation
IRB: Institutional Review Board
LLM: large language model
OSCE: objective structured clinical examination
SP: standardized patient

## Data Availability

All survey data and a description of the generative AI chatbot are presented in this manuscript.
