# Supplementary material for: Feasibility of AI and Human Standardized Patients to Enhance Customer Discovery Communication Skills in Medical Students: Preliminary Evaluation of an Observational Cohort Study
Source: JMIR Form Res. 2026 Sep 10;10:e95225. doi: 10.2196/95225 (PMC13613044; doi:10.2196/95225)
Supplement: Supplementary file 1 [file formative_v10i1e95225_app1.docx]

**Multimedia Appendix 1.**

Entrepreneurship M1 Customer Discovery student self efficacy survey

The first set of questions deal with your interview experience with **STANDARDIZED HUMAN PATIENTS.**

**Q1) The interview instructions were clear and easy to follow.** *(Select 1)*

- Strongly disagree
- Disagree
- Neutral
- Agree
- Strongly agree

**Q2) This experience with standardized patients helped me practice my customer discovery interview skills.** *(Select 1)*

- Strongly disagree
- Disagree
- Neutral
- Agree
- Strongly agree

**Q3) This experience with standardized patients was relevant to topics discussed in lecture.** *(Select 1)*

- Strongly disagree
- Disagree
- Neutral
- Agree
- Strongly agree

**Q4) I can think of a way to use my experience with standardized patients in the conduct of my own customer discovery interviews.**

- Strongly disagree
- Disagree
- Neutral
- Agree
- Strongly agree

**Q5) The interview experience with standardized patients was helpful and can be applied to my career as a physician.** *(Select 1)*

- Strongly disagree
- Disagree
- Neutral
- Agree
- Strongly agree

**Q6) What did you like or dislike about this experience with standardized patients?**

*(Free text box available)*

**Q7) Is there anything that you would have changed?**

*(Free text box available)*

This set of questions deal with your interview experience with **an AI CHATBOT STANDARDIZED PATIENT**

**Q8) The interview instructions were clear and easy to follow.** *(Select 1)*

- Strongly disagree
- Disagree
- Neutral
- Agree
- Strongly agree

**Q9) This experience with an AI chatbot standardized patient helped me practice my customer discovery interview skills.** *(Select 1)*

- Strongly disagree
- Disagree
- Neutral
- Agree
- Strongly agree

**Q10) This experience with an AI chatbot standardized patient was relevant to topics discussed in lecture.** *(Select 1)*

- Strongly disagree
- Disagree
- Neutral
- Agree
- Strongly agree

**Q11) I can think of a way to use my experience with the AI chatbot standardized patient in the conduct of my own customer discovery interviews.**

- Strongly disagree
- Disagree
- Neutral
- Agree
- Strongly agree

**Q12) The interview experience with an AI chatbot standardized patient was helpful and can be applied to my career as a physician.** *(Select 1)*

- Strongly disagree
- Disagree
- Neutral
- Agree
- Strongly agree

**Q13) What did you like or dislike about this experience with standardized patients?**

*(Free text box available)*

**Q14) Is there anything that you would have changed?**

*(Free text box available)*
