# Supplementary material for: Feasibility of AI and Human Standardized Patients to Enhance Customer Discovery Communication Skills in Medical Students: Preliminary Evaluation of an Observational Cohort Study
Source: JMIR Form Res. 2026 Sep 10;10:e95225. doi: 10.2196/95225 (PMC13613044; doi:10.2196/95225)
Supplement: Supplementary file 2 [file formative_v10i1e95225_app2.docx]

**Multimedia Appendix 2. Standardized patient sample interview questions and answers.**

Today’s class simulates two different customer discovery interview scenarios. They are designed to get students familiar with the process, especially the part of conducting interviews. Conducting good, non-biased interviews is a critical part of customer discovery as this is how students test different hypotheses about your business model canvas. The better they are at interviewing, the better they are at learning about the commercial viability of your invention.

Please refer to the rating rubric to see the characteristics of a successful customer discovery interview. The questions and qualities of a good interview should be universal – that is, the specific disease indication, technology, and scenario matter much less than the process.

Please refer to the student handout for a schedule of the day and links to videos showing the do’s / don’ts of customer discovery.

Student should describe the high-level background/purpose of the interview to the person being interviewed…I’m a student taking part in a project trying to understand how care is currently provided for XXX. By understanding the current process we hope to design better solutions.

General guidance-student should:

- Ask open-ended questions
- Ask about processes
- Ask about actions
- Ask why
- Ask what and how
- Focus on past behavior

Should avoid:

- Asking for opinions
- Asking customers to speculate
- “Would you …” questions
- Leading questions
- Multiple choice questions

Scenarios with mock scripts

A. You are a student working with a faculty mentor who is developing a software app to help patients monitor their cardiovascular disease symptoms at home in between visits to the doctor.

You are interviewing patients with cardiovascular disease who may benefit from using this app. You want to understand how they currently manage their disease, what kinds of tech they use at home, how they communicate with their provider, and if they would be comfortable using tech to monitor, report, and act on their health condition.

Here’s a sample list of good questions. The answers really don’t matter – they can be made up. The important thing is that the student gets comfortable asking a series of insightful questions, and if the conversation gets off track, how do they steer things back?

- Tell me about what kind of cardiovascular disease you have.
  - I have A fib
  - How do you manage your disease on a daily basis?
    - I take some medication, try to watch what I eat, and try to exercise. I don’t really think about it that much except when I’m in pain.
- Tell me a little more about your medications. How many do you take a day? Do you ever have trouble tracking whether you’ve taken your medicine?
  - I take 2 medicines daily, 1 medicine twice a day, and 1 medicine every other day. Yes it’s a hassle to manage everything but I think I have a good system. I use one of those pill boxes.
  - You said you don’t think about your disease that much except when you’re in pain. How often does that occur?
    - Oh maybe a couple times a week.
    - How bad is it, and what do you do about it?
      - Usually it’s not so bad, I just stop what I’m doing and sit down. If it’s really bad I might lay down and rest.
      - Do you ever have to call your doctor?
      - Once in a while, but I usually don’t bother…
  - **Student should dig deeper into anything that sounds particularly interesting,** **painful,** or **expensive…why don’t you bother?** (They don’t get back to me or by the time they get back to me I feel better)
  - Do you ever have to immediately need to go to the doctor or emergency room?
    - Maybe once or twice a year
    - **Student should dig deeper into anything that sounds particularly interesting,** **painful,** or **expensive…tell me more about your trips to the ER, etc**
- What are **top 3 challenges** you face how you manage your A Fib?
  - I can’t tell when an attack will come on, I can’t get help when I need it, I have a hard time remembering what medicines to take
- ‘let me make sure I understand your problem’ – student summarizes the situation.
  - SP agrees/disagrees based on discussion so far.
- How have you dealt with or solved your challenges managing your A Fib?
  - I try to keep a journal of my medications but I usually lose it somewhere in the house. I wish I could have someone to help me with small questions I have about my disease when something flares up, but the current system is no good.
  - Why do you lose your journal around the house? Why is the current situation no good?
  - I’m forgetful…I can’t get help when I need it
- Do you use technology like a smartphone, IPad, or computer around the house?
  - I have a computer and a smartphone
- How comfortable do you feel using this kind of technology?
  - I use my computer for email and surfing the web, usually the same for my phone, plus I play some games and read the newspaper
- Do you use apps on your phone?
  - Yes but I don’t really know how to use them
- Do you use technology to log purchases, exercise, or anything else you do on a regular basis?
  - Not really. I don’t track anything on my phone- too much hassle and I don’t want my info getting stolen.
- Do you pay for these services? Would you?
  - No way
- Ask ‘why?’ to follow up any points that are unclear or bring out particularly strong emotions
- Is there anyone else with A fib that might be interested in talking to me?
  - I have a neighbor and a cousin with A fib, let me give you their names and phone numbers
- What didn’t I ask you that I should have about you and your a fib?
  - My medications are really expensive and a pain to pick up. It would be nice if they didn’t cost so much and were easier to get.

B. You are a student working with a faculty mentor who is developing a patient/provider messaging systems for diabetes patients. You are interviewing providers who may use this system. You want to understand how they currently manage patients, what kinds of tech they use in their clinic, how they currently communicate with their patients, and if they would be comfortable using tech to remotely monitor, report, and act on their patients’ health condition.

Here’s a sample list of good questions. The answers really don’t matter – they can be made up. The important thing is that the student gets comfortable asking a series of insightful questions, and if the conversation gets off track, how do they steer things back?

- Tell me about your role at your clinic?
  - I’m an attending at a busy academic medical center
- How do you **fit** into your company’s department **structure**?
  - I’m one of 30 doctors in our clinic. I see patients 3 days a week and teach the other 2
  - What is a typical day like on your job? Dig deeper into anything that sounds painful or expensive
    - A typical clinic is 4 hrs long and I see 4 patients an hour. I also have to chart every patient and follow up with test results and other things that pop up
- What are top 3 challenges you face in your job?
  - Not enough time in the day, too much data, patients who don’t follow instructions/their treatment plans
- What are top 3 challenges you face in your job related to managing patients with diabetes?
- What is the biggest issue with the current process?
- How much time do you spend solving it?
- How have you dealt with or solved communication with patients in between visits?
  - We have MyChart
- How much do you pay for the current solution? Do you have authority to purchase anything for your clinic?
  - I don’t know. I don’t deal with financial issues in the department
- Have you tried any new technologies recently? Ask for a story
  - We have vendors come in here all the time trying to sell their products. I think the closed loop systems that Abbvie and other companies have right now are pretty good. I imagine the best thing would be to try to incorporate new tech into those existing platforms. My Chart is also big with our patients.
- Do you have the ability to start using new technology with your patients, or if not what is the approval process like?
  - We are always talking about what might be best for our patients. We brainstorm different programs in our faculty meetings all the time. We have to meet quality metrics passed down from hospital administration or our rankings on CMS and hospital websites go down.
- Ask ‘why?’ to follow up any points that are unclear or bring out particularly strong emotions
- What did you mean when you said____?
- Who else should I speak to?
- What didn’t I ask you that I should have?
