# Supplementary material for: Feasibility of AI and Human Standardized Patients to Enhance Customer Discovery Communication Skills in Medical Students: Preliminary Evaluation of an Observational Cohort Study
Source: JMIR Form Res. 2026 Sep 10;10:e95225. doi: 10.2196/95225 (PMC13613044; doi:10.2196/95225)
Supplement: Supplementary file 3 [file formative_v10i1e95225_app3.docx]

**Multimedia Appendix 3**. Customer discovery interview practice rating rubric.

|  | 0 points | 1 point | 2 points |
| --- | --- | --- | --- |
| Interview conduct | | | |
| Did the interviewer use open ended, hypothesis-driven questions? They did not sell their product, they were not asking leading questions, etc. Did the interviewer greet the interviewee and introduce themselves. Did the interviewer start with background and biography questions to establish rapport? Examples of background questions are - Who are you? What’s your role? What does a typical week look like for you? What responsibilities do you have?) (2 pts) | *Little or no use of open-ended questions (under 25%). Frequent use of leading questions and pitching of technology. Very little (under 25%) questions about background/biography. Interviewer fails to show interest in background.* | *Moderate use of open-ended questions (25-75%). Some leading questions and selling of technology. Some questions about background/biography (25 – 75%). Interviewer could ask about background more.* | *Most or all questions are open-ended (more than 75%). Little or no leading questions and selling technology. Interviewer starts with background/biography questions (more than 75%) Interviewer shows curiosity about day-to-day workflow and problems.* |
| Did the interviewer provide an intro and conclusion framing the purpose of the interview? Did they show active listening, respectful and professional behavior throughout interview? Did the interview only discuss non-confidential information? (2pts) | *Purpose of interview not explained at beginning and end of interview. Interviewer has no eye contact, does not ask follow up questions, is rude or impolite.* | *Purpose of interview partially explained at beginning and end of interview. Interviewer has some eye contact, asks some follow up questions, and is polite some of the time.* | *Purpose of interview explained at beginning and end of interview. Interviewer greets the interviewee and introduces themselves. Maintains eye contact, asks frequent follow up questions, and is polite most or all of the time.* |
| Interview content | | | |
| Did the interview identify the interest level and customer architype of the interviewee? (2pts) | *Little to no discovery of customer interest. Unclear customer architype.* | *Interviewer gains some information about level of customer interest and how they fit into the product ecosystem.* | *Interviewer has a clear understanding of customer interest and architype.* |
| Did the interview identify how the customer currently does things? (2pts) | *Interviewer asks few if any questions about current workflows. Little to no follow up questions.* | *Interviewer asks some questions about current workflows. Occasional follow up questions to learn more.* | *Interviewer asks detailed questions with follow-up about current workflows.* |
| Did the interview identify the most urgent customer problems/needs? (2pts) | *Interviewer gets little to no information about customer problems and needs.* | *Interviewers learns some information about problems and needs. Some discussion about urgency.* | *Interviewer has a thorough understanding of customer problems/needs. Level of urgency clearly identified.* |
